# Conservative vs surgical treatment of midshaft clavicular fractures: a systematic review

**DOI:** 10.1530/EOR-2025-0005

**Published:** 2026-04-07

**Authors:** Andreas Papaleontiou, Amelia Wild, Andrea Poupard, Panteleimon Tsantanis

**Affiliations:** ^1^ Trauma and Orthopaedics Resident – General Hospital Asklepieio Voulas, Athens, Greece; ^2^ Medical Student – University of Birmingham, Birmingham, England, UK; ^3^ Trauma and Orthopaedics Consultant – University Hospitals Birmingham NHS Foundation Trust, Trauma and Orthopaedics, Birmingham, England, UK

**Keywords:** clavicle fractures, midshaft clavicle fractures, orthopaedics, trauma, systematic review, orthopaedic surgery

## Abstract

**Purpose:**

- This systematic review aimed to evaluate the effectiveness of surgical versus conservative management in terms of union rates, functional outcomes and complications.

**Methods:**

- A systematic review was conducted according to the Preferred Reporting Items for Systematic Reviews and Meta-Analyses (PRISMA) guidelines. Randomised controlled trials published between January 2017 and November 2023 were sourced from MEDLINE, Embase, PubMed and Cochrane databases. Outcomes assessed included union rates, functional scores and complications.

**Results:**

- Six trials, involving 579 patients with midshaft clavicle fractures, met the inclusion criteria. The union rate was higher in the surgical group (93.3–100%) compared to the conservative group (82–94%). Time to union was significantly shorter for surgical management in two out of three studies reporting this outcome. Shoulder scores revealed short-term improvements in surgical groups but no significant differences at 12 months or longer. Complication rates were comparable: hardware irritation was more frequent in surgical groups, while non-union was more prevalent in conservative management. Only two studies found a statistically significant difference in union rates favouring surgery.

**Conclusion:**

- Surgical management offers higher union rates and faster recovery but does not demonstrate significant long-term advantages in functional outcomes compared to conservative treatment. Complication rates are similar, with distinct profiles. Clinicians should prioritise patient-centred decision-making, considering individual preferences and clinical contexts, as operative intervention may not significantly enhance long-term outcomes. Further research should perform subgroup analyses to refine management strategies.

## Introduction and background

The midshaft is the most frequently injured site of clavicular fractures, accounting for 75–80% of cases (1). While 2.6–4% of adult fractures are clavicle fractures, clavicle fractures are more prevalent in young and active individuals and are mostly sustained from falls onto the lateral aspect of the shoulder (2).

These fractures can be classified as displaced or non-displaced and as type A, type B and type C as per AO classification. The AO classification is summarised in Table 1 (3).

**Table 1:** AO fracture and dislocation classification (3).

Type A = simple
A1 = spiral
A2 = oblique
A3 = transverse
Type B = wedge
B1 = spiral wedge
B2 = bending wedge
B3 = fragmented wedge
Type C = complex
C1 = complex spiral
C2 = segmental
C3 = irregular

Surgical management is predominantly open reduction internal fixation (ORIF) with a plate or, less frequently, intramedullary nail fixation. The literature indicates no significant difference in outcomes between the two methods of fixation (4, 5), although one study indicated that the rate of implant removal for intramedullary nails was almost two times the rate for the plate fixation group (38 vs 73%) (4).

Non-surgical management options include the use of a sling or ‘figure-of-eight’ bandage, in combination with rehabilitation. Rehabilitation initially includes passive range of movement exercises, followed by strengthening exercises until return to normal activity, including sports, can be recommended. Typically, recovery and return to sport is expected at about 4–6 months post-injury (6).

As per current practices, absolute indications for surgical management include open fractures, neurovascular injury, skin tenting due to displacement and floating shoulder (7). Type C fractures per AO classification are also usually treated surgically. Complications of surgical management include the need for reoperation, infection, hardware irritation, neurovascular injury and pneumothorax. Complications of non-surgical management include non-union/malunion, poor aesthetic and decreased shoulder strength.

Currently, no clear consensus prevails on the management of midshaft clavicle fractures that do not meet absolute indications for operation. In clinical practice, young and active individuals typically receive an operation, but the current evidence for the benefits of operative management is not strong. Furthermore, good recovery from non-operative management has been reported, indicating the possibility of current overtreatment. Conversely, surgical management offers the benefits of earlier recovery and regain of pre-injury function. While some studies indicate higher shoulder scores in surgical groups, others indicate no significant difference. Thus, current evidence indicating preferential management options is conflicting (5, 7, 8).

The last review of randomised controlled trials (RCTs) investigating conservative vs surgical management of midshaft clavicle fractures was performed in 2021 (8). This review included patients with intramedullary nail fixation in addition to plate fixation, which is a less commonly used technique. In addition, numerous RCTs have been published since this date, and therefore, an update to the evidence base is required.

### Aim

The primary aim of this systematic review is to investigate whether surgical management of midshaft clavicular fractures is superior to non-surgical treatment in terms of union, shoulder function and complications.

## Methods

The main objectives of this review are as follows:

- To establish the outcomes of displaced clavicle fractures with surgical management compared to those managed with non-surgical management, relating to union and functional scoring.
- To compare the complications associated with displaced clavicle fractures with surgical management compared to those managed non-surgically.
- To establish whether surgical management of displaced clavicle fractures is preferable to non-surgical management in practice.

### Search strategy

A systematic literature review was conducted in November 2023, in accordance with the Preferred Reporting Items for Systematic Reviews and Meta-Analyses (PRISMA) guidelines, on the following databases: MEDLINE, Embase, PubMed and Cochrane database of controlled trials. The search was limited to studies published in English from January 2017 to November 2023. Two searches were performed by two separate authors on separate dates.

Subject headings (MeSH terms) and keywords were used surrounding the concepts of ‘clavicle’, ‘midshaft’ and ‘fracture’, which were combined with Boolean operators. Citations of included studies and relevant systematic reviews were also searched for additional papers.

Duplicates were manually removed, and title and abstract screening was undertaken. This was completed by two independent reviewers, with any disputes resolved through discussion with the remaining two authors. Full-text screening was carried out according to the pre-defined inclusion and exclusion criteria (Table 2). Full texts were obtained through university access or by directly contacting authors. Texts that had not been able to be accessed after 2 months were excluded from the review.

**Table 2:** Inclusion and exclusion criteria.

	Inclusion criteria	Exclusion criteria
Population	Adult populations (>18 years old)	Skeletally immature patients
	Patients with a displaced midshaft clavicle fracture, as determined according to the AO fracture classification criteria (Table 1). All degrees of displacement included.	Paediatric populations (<18 years old)
Intervention	Surgical interventions: plate fixation	Studies in which results for surgical interventions are grouped, not enabling extrapolation of results among surgical interventions.
		Surgical management using intramedullary nail fixation.
Comparator	Any non-surgical interventions	
Outcome	Functional shoulder scoring (any, including DASH and Constant–Murley scores)	
	Union/non-union rates	
	Complications (including infection, paraesthesia and reoperation)	
Language	Publications in English	Non-English publications
Study design	Randomised controlled trials	Retrospective studies
	Prospective observational trials	Case series/reports
		Systematic reviews, meta-analyses and literature reviews
		Conference abstracts
Others	Published from January 2017 to October 2023	Papers published outside of January 2017–October 2023

Eligible studies must recruit patients older than 18 years with a recently diagnosed isolated midshaft clavicular fracture of any displacement grade, managed either surgically or non-surgically. The surgical intervention included plating, and the non-surgical included immobilisation and rehabilitation.

Case reports, review articles, letters and conference abstracts were excluded. Studies considering data comparing operative management of displaced midshaft clavicle fractures in adult populations were included.

### Data extraction

The following data were extracted from each study onto a pre-prepared data form: study design, sample size, patient demographics (age and sex), method of management (surgical technique and conservative management protocol), length of follow-up and outcomes (union rate, shoulder score and complications). Mean differences and median values were calculated using these data.

### Risk of bias

Risk-of-bias assessment was undertaken for each included study according to study type. The Cochrane Collaboration Risk of Bias 2 tool was used for randomised controlled trials, and the Risk of Bias In Non-randomized Studies of Interventions was used for non-randomised trials.

This review was not registered, and no protocol was prepared.

## Results

**Figure 1:**
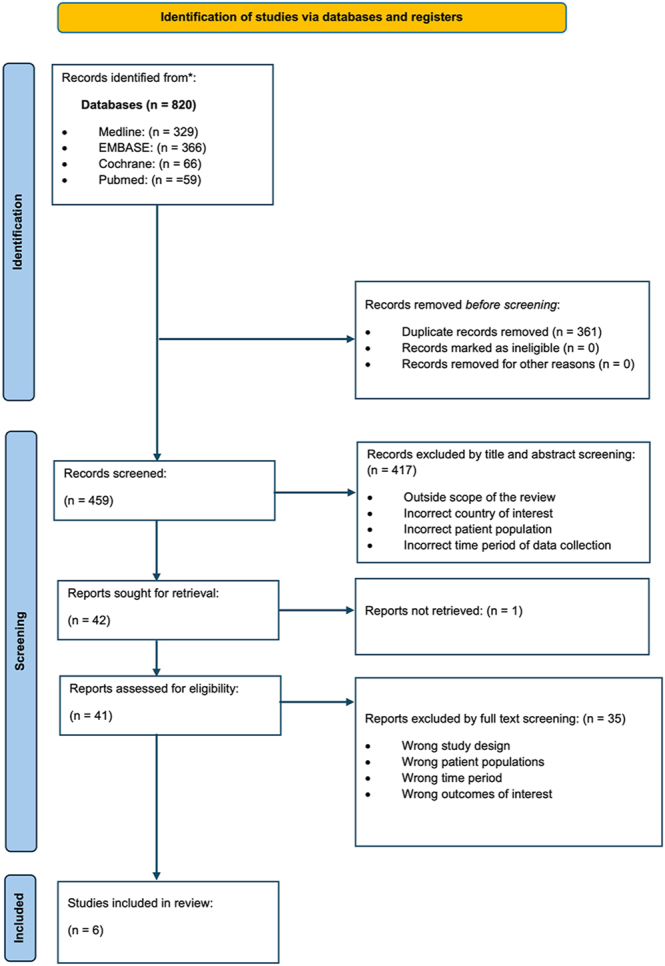
PRISMA flow diagram of study selection process.

The search resulted in a total of 820 records being identified. No studies were identified from citation screening. A total of 459 records underwent title and abstract screening, and after full-text screening of 41 records, 6 randomised controlled trials met the inclusion criteria. The complete study selection process is shown in Fig. 1.

Overall, a total of 579 midshaft clavicular fractures are included in the review, with 300 (51.8%) patients being treated operatively and 279 (48.2%) being treated conservatively. The mean age ranged from 31.9 to 40.3. The mean age of patients treated surgically was 36.19 and the mean age of those treated non-surgically was 43.58 across the five studies that reported the mean age. The analysis includes 442 men and 137 women. Selected studies and characteristics are detailed in Table 3.

**Table 3:** Study and participant characteristics of included studies.

Study	Number of patients			Gender		Mean age, years	
	Total	SG group	Non-SG group	Male	Female	SG group	Non-SG group
Ban *et al.* (9)	120	60	60	101	19	37.5 (18–51)	39 (19–63)
Qvist *et al.* (10)	146	75	71	119	27	40 (18–60)	39 (18–60)
Bhardwaj *et al.* (11)	69	36	33	21	48	32.4 ± 43	31.7 ± 26
Parthak *et al.* (12)	42	18	24	30	12	38.33	40.3
Kumar *et al.* (13)	40	25	15	31	9	18–60; 31–40*	31–40*
Nicholson *et al.* (14)							
RCT	162	86	76	140	22	32.7	31.9
PCS	226	0	200	149	51	-	36

SG, surgical; RCT, randomised controlled trial; and PCS, prospective cohort study.

* Median value.

Non-surgical management was similar in each study, comprising immobilisation for 3–4 weeks in a sling, figure-of-eight bandage or arm pouch. The outcomes measured included shoulder function using the Constant–Murley score (CMS) (*n* = 3), the Disabilities of the Arm, Shoulder, and Hand (DASH) score (*n* = 5), the American Shoulder and Elbow Surgeons (ASES) Standardized Shoulder Assessment Form (*n* = 1) and the University of California Los Angeles (UCLA) score (*n* = 1). In addition, union/non-union rates were investigated and complications including infection, paraesthesia and reoperation were reported in all studies. The shortest follow-up period was 6 months, and the maximum was 24 months. The management details, outcomes and follow-up duration of each group are given in Table 4.

**Table 4:** Summary of interventions and measured outcomes for included studies.

Study	Surgical treatment	Non-surgical treatment	Outcomes measured and methods measuring them	Follow-up duration
Ban *et al.* (9)	Anatomical locking plate within 3 weeks of injury	Sling for 2 weeks	Danish version of the Constant–Murley score, DASH score, radiological union and complications	At 6 weeks and at 6 and 12 months
Qvist *et al.* (10)	Pre-contoured plate and locking screws within 2 weeks of injury	Sling – maximum of 3 weeks. Encouraged to discontinue the sling when they no longer felt it was necessary and to use the arm and shoulder within the limits of pain. No physiotherapy	DASH score, the Constant–Murley score, radiographic evidence of union and complications	At 6 weeks and after 3, 6 and 12 months
Bhardwaj *et al.* (11)	3.5 mm LC pre-contoured plate applied superiorly	Arm pouch 4 weeks; then assisted passive mobilisation. No strenuous work for at least 3 months	Functional outcome (Constant–Murley score), non-union, malunion, plate prominence and superficial infection	At 1, 3, 6, 12 and 24 months; 100% of patients at follow-up
Parthak *et al.* (12)	Superior surface titanium anatomical locking plate applied superiorly within 10 days of injury. Two-week immobilisation and then similar rehabilitation as non-operative cohort	Figure-of-eight bandage immobilisation. Pendulum and Codman exercises performed for the 3 weeks followed by restricted active shoulder abduction and adduction exercises for 3 weeks. Full range of motion exercises at 6 weeks	DASH and ASES scores, patient’s satisfaction, union and complications	At 6 weeks and at 3, 6 and 12 months
Kumar *et al.* (13)	Pre-contoured locking compression plate	Figure-of-eight clavicular braces for 4 weeks	Quick DASH score, UCLA, x-rays to check for implant position, malunion, radiological union and complications	At 6 weeks and at 3, 6, 9 and 12 months
Nicholson *et al.* (14)	Plate – unspecified. Operated within 2 weeks of injury. Rehabilitation identical to non-surgical group	Sling for 2 weeks + physiotherapy	DASH score, quick DASH, 14 and EQ-5D, displacement (x-ray) and union	At 6 and 12 weeks and at 6 months

ASES, American Shoulder and Elbow Surgeons; EQ-5D, EuroQol five-dimension summary index.

### Heterogeneity of participants and methodologies

The studies showed similar selection criteria, study designs and interventions in both groups. They had comparable age groups and higher proportions of male patients. The baseline medical health of participants was not detailed, which limits the assessment of participant homogeneity. While shoulder scores used differed in some studies, outcomes and methods of measuring the outcomes were similar across studies. Subgroup analysis was not deemed necessary given the overall similarity across study participants.

### Risk-of-bias results

**Figure 2:**
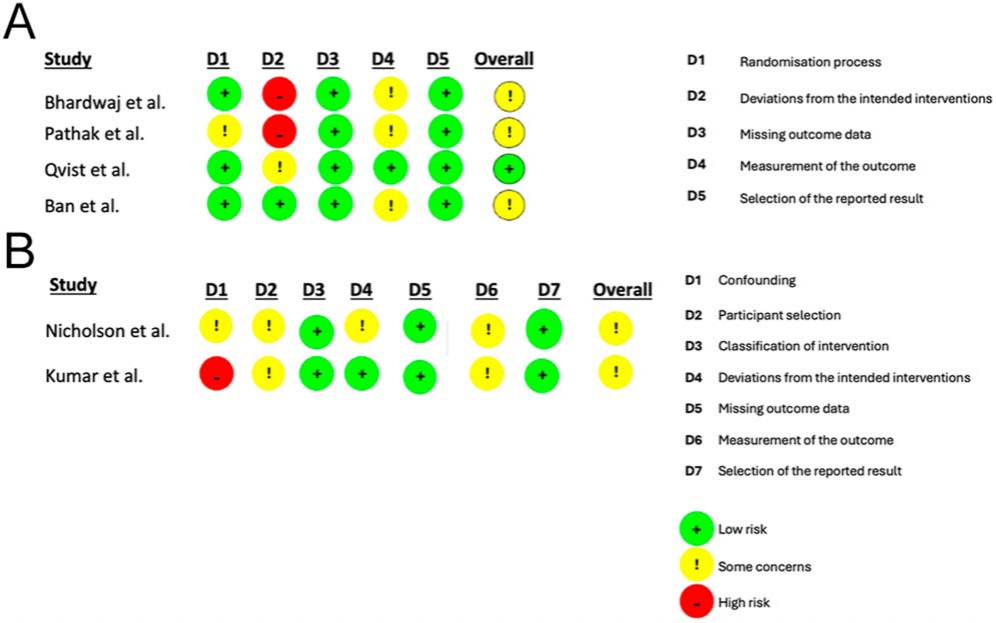
(A) Risk-of-bias assessment for randomised studies using the Cochrane RoB 2 tool. (B) Risk-of-bias assessment for non-randomised studies using the Cochrane ROBINS-I tool.

Assessment of methodological quality identified Qvist *et al.* as the only study with a low risk of bias (10). The remaining five studies had a moderate risk of bias. These five studies had concerns relating to the measurement of the outcome domain, largely due to outcome assessor awareness of which cohort the participant was part of, with no measures introduced to minimise this potential bias. Results for each domain across all studies are shown in Fig. 2.

### Union rates

Across the analysed cohorts, non-surgical strategies demonstrated varying degrees of success in achieving fracture union, with rates ranging from 82 to 94%. In comparison, the union rate in the surgical group across all studies ranged from 93.3 to 100%. All studies indicated a higher union rate in the surgical group, but only 2 of the 5 studies comparing the union rates found statistically significant differences (9, 10). Qvist *et al.* concluded a greater rate of union in the surgical group (*P* < 0.009), while Ban *et al.* identified a greater risk of symptomatic non-union in the non-operative group compared to the operative group (*P* = 0.014). The relative risk was 9.47 (95% CI: 1.26–71.53) (9). In addition, higher notable challenges, such as symptomatic non-union, were observed in the conservatively treated group, underscoring the limitations of conservative management.

The average non-union rate in non-surgical groups was 13.2%, while in surgical groups, it was 2.3%. Ban *et al.* reported that 18% of patients from the non-operative cohort failed to achieve radiological fracture union at six months; this was the highest non-union rate across studies (9). Notably, eight of the patients who did not achieve union experienced pain, and functional decline, prompting their transition to open reduction and plate fixation. At the 12-month follow-up, all eight patients who required operation exhibited bone union. Conversely, the remaining two non-operative patients who experienced non-union were asymptomatic and exhibited normal function at 6 and 12 months. Qvist *et al.* reported that of the 11 non-operative patients who developed non-union, 1 was asymptomatic and 10 were symptomatic. Nine of these were operated, and all achieved union at 12 months (10).

Three studies reported the mean union time (11, 12, 13). Parthak *et al.* reported a mean union time of 14.21 weeks in the non-operative group and 12.38 weeks in the operative group (*P* = 0.02) (12). Union was seen at 15.6 ± 0.8 weeks in the operative group and at 22.8 ± 0.4 (*P* < 0.0001) weeks in the non-operative group in the study of Bhardwaj *et al.* (11) In Kumar *et al.*’s study, 28% of non-operative patients reached fracture union within less than 12 weeks, whereas 60% of operated patients reached union in under 12 weeks. Despite that, Kumar *et al.* indicated no significant difference in union rate between the two groups at the 1-year follow-up (92% union rate in the non-operative group and 93.3% union rate in the operative group) (13). Table 5 summarises the union and non-union rates across studies, alongside *P*-value for non-union between the two groups.

**Table 5:** Summary of union and non-union rates in surgical and non-surgical groups across studies. *P* value reported for non-union between two groups. Statistically significant results are in bold.

Study/intervention	Union rate	Non-union rate	***P*** value
Ban *et al.* (9)			**0.014** *
Non-operative	82%	18%	
Operative	98%	2%	
Qvist *et al.* (10)			
Non-operative	83%	17%	**0.009**
Operative	97%	3%	/
Bhardwaj. *et al.* (11)			
Non-operative	94%	6%	0.4501
Operative	100%	0%	/
Pathak *et al.* (12)			
Non-operative	91.7%	8.3%	0.16
Operative	100%	0%	/
Kumar *et al.* (13)			NS
Non-operative	92%	8%	
Operative	93.3%	6.7%	
Nicholson *et al.* (14)			
Non-operative	86.5%	13.5%	/

* Symptomatic non-union.

NS, not significant.

### Shoulder scores

Six studies commented on the functional outcomes post-injury using different scoring systems, namely CMS, DASH, ASES and UCLA. Selected scoring systems varied across studies, with most using two. However, the multiplicity of scoring tools with patient-reported outcomes contributes to the study heterogeneity and limits their comparability.

Five studies evaluating outcomes with the DASH score presented comparable results: the differences between the operative and non-operative cohorts were statistically insignificant after a minimum of 6 months of follow-up (9, 10, 12, 13, 14). Statistical significance in the DASH score was observed between the two groups in two studies at 6 weeks and 3 months of follow-up, but this difference was not maintained beyond 6 months (9, 10). Three studies using the CMS score found different results. Bhardwaj *et al.* stated a statistically significant difference between the two groups at 24 months of follow-up (*P* < 0.0001), while Qvist *et al.* and Pathak *et al.* stated statistically different results between the two interventions below 6 months of follow-up (9, 10, 11). Pathak *et al.* used the ASES score and reported statistical differences in outcomes at the 3- and 6-month follow-ups that faded in the longer term (12). The last score used, UCLA scale, in one paper reported equivalent results, i.e. the lack of statistical differences between the two groups at any point of the follow-up period (13).

The majority of the studies followed up the two cohorts with functional shoulder scoring at multiple time intervals, ranging from 6 weeks to 24 months. Four studies assessed functional outcomes at 6 weeks, two of which identified a significant improvement for surgical treatment compared to non-surgical (*P* < 0.001) (9, 10). In comparison, Nicholson *et al.* and Pathak *et al.* did not identify a significant difference in shoulder scoring at any follow-up time, including <6 weeks. Qvist *et al.* identified a significant difference (*P* < 0.02) at the 3-month follow-up in terms of DASH and CMS scores, again favouring surgical management (10). Of three other studies assessing shoulder scores at this time point, none identified a significant difference (12, 13, 14). Of the studies that followed up with functional shoulder scores at 6, 9 or 12 months, none found significant differences at these stages of follow-up (9, 10, 12, 13, 14).

Bhardwaj *et al.* is the only study in which scores for multiple follow-up periods were not reported, which limits the interpretation of the results (11). This study reported that the CMS score was significantly better at the 24-month follow-up for surgical patients compared to non-surgical (76.24 compared to 89.42, *P* = <0.0001).

At the 12-month follow-up, no statistical difference in shoulder score was reported between the groups. Table 6 summarises the functional outcomes.

**Table 6:** Functional outcome scores of surgical and non-surgical techniques, measured using DASH, CMS, ASES and UCLA scoring systems. Results displayed for all follow-up periods reported. Data are presented as mean (SD) or range. Statistically significant results are in bold.

Study/functional score/FU period	Nonoperative	Operative	***P*** value
Ban *et al.* (9)			
DASH			
6 weeks	22 (1–76)	10 (0–59)	**<0.001**
6 months	7 (0–51)	3 (0–38)	0.145
12 months	3 (0–73)	0 (0–22)	0.277
CMS			
6 months	92 (36–100)	94 (46–100)	0.185
12 months	96 (72–100)	96 (72–100)	0.184
Qvist *et al.* (10)			
DASH			
6 weeks	25 (13–40)	14 (3–24)	**<0.001**
3 months	7 (3–25)	2 (0–15)	**0.02**
6 months	4 (0–13)	3.8 (0–10)	>0.05
12 months	1 (0–5)	1 (0–10)	>0.05
CMS			
6 weeks	75 (55–89)	90 (68–95)	**<0.001**
3 months	90 (79–100)	97 (85–100)	**0.02**
6 months	95 (85–100)	96 (87–100)	>0.05
12 months	98 (87–100)	97 (86–100)	>0.05
Bhardwaj *et al.* (11)			
CMS			
24 months	76.24 (3.43)	89.42 (5.61)	**<0.0001**
Pathak *et al.* (12)			
DASH			
6 weeks	37.69 (4.89)	35.93 (4.59)	0.254
3 months	18.73 (4.53)	17.61 (3.54)	0.507
6 months	11.61 (3.78)	10.40 (3.04)	0.319
12 months	7.27 (2.92)	6.30 (2.64)	0.191
ASES			
6 weeks	69.07 (5.69)	72.10 (6.69)	0.101
3 months	77.99 (5.19)	82.46 (6.27)	0.028
6 months	85.86 (5.03)	90.01(5.11)	0.022
12 months	93.39 (4.10)	94.55 (55.97)	0.178
Kumar *et al.* (13)			
DASH			
3 months	35.18 (14.23)	38.94 (20.42)	0.726
6 months	21.16 (12.37)	25.11 (17.41)	0.989
9 months	4.63 (6.07)	9.18 (12.19)	0.709
12 months	0.28 (0.76)	1.37 (3.51)	0.219
UCLA			
3 months	21.76 (6.57)	19.87 (8.47)	0.726
6 months	26.76 (6.7)	26 (8.19)	0.989
9 months	31.12 (4.67)	29.8 (6.19)	0.709
12 months	34.16 (2.32)	32.8 (3.95)	0.219
Nicholson *et al.* (14)			
DASH			
6 weeks	25.5 (21.2–29.9)	22.9 (19–29)	0.385
3 months	11.8 (8.2–15.3)	10.4 (7.7–13.1)	0.532
6 months	5.8 (3.0–8.6)	5.2 (3.4–7.1)	0.701

FU, follow-up.

### Complications

Five studies reported findings on complications. Kumar *et al.* reported that the number of patients with complications varied significantly between the operative (9/15) and non-operative (5/25) groups (*P* = 0.01), although no significance testing was completed for mal- or non-union rates specifically, potentially due to small sample sizes (13). In comparison, studies by Pathak *et al.* and Bhardwaj *et al.* reported no significant differences between groups for occurrence of non- and malunion (11, 12). Pathak *et al.* identified that the only reported complication that did show a significant difference between groups was hardware irritation (*P* = 0.04) (12).

Infection was identified in one operative patient in Bhardwaj *et al.* and in one operative patient in Kumar *et al.*, but neither were significantly different rates to the non-operative groups (11, 13). Ban *et al.* identified various complications in 26% of the operative (*n* = 14), leading to surgery, and in 26% of the non-operative groups (*n* = 15) (9). Such complications include symptomatic non-union, bony prominences and irritation at the implant site. However, no significant differences were observed for these complications between groups. Nicholson *et al.* reported one patient with adhesive capsulitis and another with a symptomatic bony prominence; however, no detailed analysis according to the patient group was completed (14). Rates of complications, and significant values where reported, are given in Table 7.

**Table 7:** Summary table of reported complications. The number of patients with complications is presented. Statistically significant results are in bold.

Study/complication	Operative	Non-operative	***P*** values
Ban *et al.* (9)			
Non-union	1	10	NS
Symptomatic		8	
Asymptomatic		2	
Malunion	-	-	
Other complications			0.865*
Adhesive capsulitis	1	1	
Required second operation	14	15	
Infection	0	0	
Bony prominence		1	
Qvist *et al.* (10)			
Non-union	2	11	**0.009**
Malunion	-	-	
Other complications			
Dysaesthesia at site	44 (70%)	13 (21%)	
Failure of screw fixation	1		
Bhardwaj *et al.* (11)			
Non-union	0	2	0.4501
Malunion	1	3	0.5491
Other complications			
Plate prominence	2	0	0.999
Infection	1	0	0.5371
Pathak *et al.* (12)			
Non-union	0	2 (8.33)	0.16
Malunion	0	3 (12.5)	0.08
Other complications			
Paraesthesia	3	0	0.082
Infection	1	0	0.33
Shortening	0	2	0.16
Hardware irritation	4		**0.04**
Kumar *et al.* (13)			
Non-union	2	2	
Malunion	2	1	
Other complications			
All	9	5	**0.01**
Nicholson *et al.* (14)			
Non-union	-	-	
Malunion	-	-	
Other complications	-	-	

* Comparing all complications.

## Discussion

This systematic review aimed to evaluate the efficacy of surgical management of midshaft clavicle fractures, compared to non-surgical methods. The evidence presented in this review suggests that union rates may be greater using surgical methods, while complication rates were minimally affected by the choice of management. Shoulder scores appeared to show preferential results towards surgical options in the short term, but this disparity minimises with continued follow-up.

This systematic review identified a greater average rate of non-union in conservatively managed groups across the studies included in this review (13.2% for non-surgical compared to 2.3% for surgical), with all studies reporting absolute values of greater union in the surgical groups. Comparable findings can be found elsewhere in the literature: Axelrod *et al.* conducted a systematic review and meta-analysis of 22 RCTs in 2020, which identified similar rates of union to those found in this systematic review: operative: 96.7%; non-operative: 88.9% (*P* < 0.001) (15). Guerra *et al.* similarly identified a difference in union rates, with surgical options showing a significantly lower risk ratio of non-union (0.10, *P* < 0.001) (16). Guerra *et al.* did include patients with intramedullary nail fixing, but this did not show any significant difference in union rates to those of plate fixation (16).

In this systematic review, the average non-union rate in the non-surgical group was 13.2% and it was 2.3% in the surgical group. All studies indicated a higher union rate in the surgical group, but only two out of five studies comparing the union rates found a statistically significant difference in either non-union or symptomatic non-union (Ban *et al.* (9) and Qvist *et al.* (10)). In addition, where reported, time to union varied among studies in this review. Out of three studies reporting time to union, two studies found a significantly shorter time in the surgical group (11, 12). Kumar *et al.* found that 32% more patients in the operative group reached fracture union under 12 weeks compared to the non-operative group (13). While this difference is statistically significant, the clinical relevance of these findings remains undetermined, particularly as no significant differences in union rates were observed at the 12-month follow up. Therefore, it is possible that non-operative patients achieved fracture union a number of weeks after the operative patients, without affecting the overall recovery and outcome. While certain evidence presented in this review suggests preference of surgical management for union rates, the clinical significance of this difference remains undetermined, particularly as there was limited evidence on the time to union and the long-term effects of this. This study highlights the need for a multi-factorial and clinical-centred approach to considering results regarding rates and time to union.

Shoulder scores are an important metric of functional improvement, critical in assessing patient satisfaction. Three studies in this review identified a significant difference in either DASH or CMS scores, favouring surgical groups (9, 10, 11). For certain patients, such as those who are independent or have an active lifestyle, a quicker functional recovery may prioritise and indicate a preferred management technique. Of four studies with a minimum follow-up period of 6 weeks, only two found significantly superior shoulder scores with operative management. However, any differences observed were not sustained in the longer term, with only the study by Bhardwaj *et al.*, which included 70 patients, identifying a significant difference in shoulder scores beyond 3 months (11). Very short-term outcomes have not been widely assessed outside of this review. In a previous systematic review, a faster return to activities was reported in the surgical group, although this was found not to be statistically significant, while other reviews focus on shoulder scores in a follow-up period of 1 year (15, 16). No strong conclusion can be drawn regarding very short-term functional outcomes, and the implication of these results requires discussion with patients as to the priorities of their recovery, balancing a potentially quicker improvement in function with surgical risks and other complications.

Moreover, when considering the DASH score after 6 weeks, the majority of cases in this review identified that function was restored with minimal impairment (DASH < 10) for both cohorts, so although differences in the absolute scores could be observed in some cases, the clinical differences and patient experience may not be distinguishable. 

A previous systematic review reported similar findings, with minimally better results in DASH and CMS score in the operative group, but not meeting the minimal clinically important difference (MCID) threshold for a significant improvement in functional outcome (15). This highlights that although some studies in this review reported better shoulder scores for the operative group, patient experience may not reflect these theoretical differences. It is important to counsel patients on their choice of management, taking into account speed of return to function, and balance this with the potential risks of each method, including surgical risks and other complications discussed in this review.

This review identified few significant differences in complication rates between operative and non-operative treatments for midshaft displaced clavicle fractures, although small absolute numbers may affect this statistical testing. These findings reinforce those of a previous systematic review that assessed 20 studies comparing non-operative management to ORIF and to intramedullary nailing (17). The network meta-analysis in this review similarly identified no statistical differences in rates of major complications between ORIF and non-surgical intervention, while studies in the current review identified no significant differences in re-operation rates (17). Guerra *et al.* identified that across 14 studies, the incidence of complications was significantly greater in the surgical group (*P* < 0.001), with 17% of patients having to undergo subsequent surgery, compared to 13% of the non-operative group (*P* = 0.02) (16). However, other studies did not align with these findings. Axelrod *et al.* identified an odds ratio of 0.85, favouring operative management for revision surgery, while Yan *et al.* identified a lower risk (risk ratio: 0.21) of bone-related major complications in the surgical group (8, 15). However, these studies did include multiple surgical techniques, rather than directly comparing plating to conservative methods.

This systematic review was subject to limitations. First, comparing surgical to conservative management inherently produces bias due to the inability to blind patients to their treatment. One study aimed to reduce further bias of making those interpreting the results blind to the management technique (10). Second, different shoulder scoring systems were used in the studies included in this review. This limited comparability across all studies. However, this was minimised as five of six studies did use the DASH score, although complete homogeneity was not achieved. Heterogeneity of studies included in this review resulted in a meta-analysis not being completed. Finally, only six studies met the inclusion criteria for this review, reducing the total number of patients being assessed and the ability to perform subgroup analysis. However, the selection criteria were specifically chosen to include only randomised controlled trials assessing the most clinically relevant techniques.

The findings of this up-to-date systematic review align with previous results. Current evidence relating to clinical implications is limited in terms of significance and highlights the importance of clinical assessment and decision-making. As a result, further evidence should prioritise subgroup analysis to identify any patient groups with better outcomes with surgical compared to non-surgical management. This will facilitate clinical discussions and enable informed decision-making.

## Conclusion

This systematic review found no significant improvement in long-term shoulder scores or complications and minimal improvements in union rates with operative management. The impact on patients of the limited observed differences between surgical and non-surgical management is unknown, particularly with consideration to similarities in functional scores. Therefore, this review underscores the importance of clinical judgement and provides a clear explanation and inclusion of patients for patient-centred decisions. Patients should be consulted that an operation is unlikely to significantly improve their function in the long term, but it might decrease the possibility of non-union.

## ICMJE Statement of Interest

No authors had competing interests when completing this review. No financial or other support was gained for this review.

## Funding Statement

This research received no specific grant from any funding agency in the public, commercial or not-for-profit sectors.

## Author contribution statement

PT conceived the idea. All authors (AP, AW, APoupard, PT) designed the study. AW and APoupard conducted the literature search and data extraction. All authors contributed to the analysis and interpretation of the data, drafted the manuscript and approved the final version for publication.
